# The host range of * Aphis gossypii* is dependent on aphid genetic background and feeding experience

**DOI:** 10.7717/peerj.7774

**Published:** 2019-09-27

**Authors:** Lin Ma, Meng-Yue Li, Chun-Yan Chang, Fang-Fang Chen, Yang Hu, Xiang-Dong Liu

**Affiliations:** Department of Entomology, Nanjing Agricultural University, Nanjing, China

**Keywords:** Cotton-melon aphid, Feeding experience, Genotype, Host range expansion, Artificial diet, Host specialization

## Abstract

**Background:**

A polyphagous insect herbivore has a wide range of host plants. However, it has been found that many polyphagous herbivores commonly exhibit a strong preference for a subset of species in their broad host range, and various host biotypes exist in herbivore populations. Nutrition and secondary metabolites in plants affect herbivore preference and performance, but it is still not clear which factors determine the host range and host preference of polyphagous herbivores.

**Method:**

Cotton-melon aphids, *Aphis gossypii* Glover, collected from cotton and cucumber crops, were used in this study. The genetic backgrounds of these aphids were detected using microsatellite PCR and six genotypes were evaluated. Performance of these six aphid genotypes on excised leaves and plants of cotton and cucumber seedlings were examined through a reciprocal transplant experiment. In order to detect whether the feeding experience on artificial diet would alter aphid host range, the six genotypes of aphids fed on artificial diet for seven days were transferred onto cotton and cucumber leaves, and then their population growth on these two host plants was surveyed.

**Results:**

Aphids from cotton and cucumber plants could not colonize the excised leaves and intact plants of cucumber and cotton seedlings, respectively. All six genotypes of aphids collected from cotton and cucumber plants could survive and produce offspring on artificial diet, which lacked plant secondary metabolites. The feeding experience on the artificial diet did not alter the ability of all six genotypes to use their native host plants. However, after feeding on this artificial diet for seven days, two aphid genotypes from cotton and one from cucumber acquired the ability to use both of the excised leaves from cucumber and cotton plants. The two aphid genotypes from cotton conditioned by the feeding experience on artificial diet and then reared on excised cucumber leaves for >12 generations still maintained the ability to use intact cotton plants but did not establish a population on cucumber plants. However, one cucumber genotype conditioned by artificial diet and then reared on excised cotton leaves could use both the intact cotton and cucumber plants, showing that the expansion of host range was mediated by feeding experience.

**Conclusion:**

Feeding experience on artificial diet induced the expansion of host range of the cucurbit-specialized *A. gossypii*, and this expansion was genotype-specific. We speculated that feeding on a constant set of host plants in the life cycle of aphids may contribute to the formation of host specialization.

## Introduction

Most insect herbivores have specialized diets (Jaenike, 1990; Via, 1991; Caballero, Ramirez & Niemeyer, 2001; Stone et al., 2009). Even polyphagous species show some degree of dietary specialization, and they often perform better on some host plants than on others. Particular insect herbivore populations may perform better on their natal hosts than on non-natal or novel hosts (Jaenike, 1990). The formation of host biotypes and the potential flexibility of dietary specialization within polyphagous species is not well understood. For example, host specialization in the polyphagous aphids, *Acyrthosiphon pisum* and *Rhopalosiphum maidis*, could not be modified by feeding experience on a novel host (Via, 1991; Caballero, Ramirez & Niemeyer, 2001). However, host specialization in other insect herbivore species was alterable (Singer, Thomas & Parmesan, 1993; Messina, Mendenhall & Jones, 2009; Wu et al., 2013). The introduction of a novel plant into a region led to a change in host availability for *Euphydryas editha* butterflies, and even resulted in a local population’s refusal to accept their ancestral hosts (Singer, Thomas & Parmesan, 1993). The seed beetles, *Callosobruchus maculatus* (F.), performed worse when they were fed on lentil than on mung bean, but after ten generations on lentil, their survival rate increased up to 88% which was similar to the value (97%) on mung bean, and consequently a self-sustaining population on lentil resulted (Messina, Mendenhall & Jones, 2009). These results illustrate that the diet of insect herbivores can change in response to resource availability (Cates, 1980).

The cotton-melon aphid, *Aphis gossypii* Glover, has a wide host range, perhaps 900 species belonging to 116 plant families, such as Cucurbitaceae, Malvaceae, Solanaceae, Rutaceae, and Asteraceae (Ebert & Cartwright, 1997; Blackman & Eastop, 2000). However, many studies illustrate that *A. gossypii* populations have formed obvious host races, which use only a subset of host plant species in their recorded host range (Kring, 1959; Guldemond, Tigges & De Vrijer, 1994; Liu, Zhai & Zhang, 2002; Liu, Zhai & Zhang, 2008; Liu et al., 2004). Numerous cases of host race, biotype, and host-specialized strains in *A. gossypii* populations have been found all over the world. In Europe, the aphid *A. gossypii* from chrysanthemum could not colonize cucumber and *vice-versa*, cucumber aphids did not use chrysanthemum (Guldemond, Tigges & De Vrijer, 1994). Five host races (cucurbits, cotton, eggplant, potato, and chili- or sweet-pepper races) were identified in *A. gossypii* populations collected from five plant families in five large geographical regions (Carletto et al., 2009). In China, *A. gossypii* populations on cotton could not colonize cucumber seedlings, and the populations collected from cucumber could not colonize cotton seedlings either (Liu, Zhai & Zhang, 2008; Wang et al., 2016). The populations on cucumber could not use chrysanthemum, but these on cotton could use this plant (Liu, Xu & Lei, 2017). These results indicate that *A. gossypii* populations have differentiated into at least two host biotypes: cotton- and cucurbit-specialized aphids.

Moreover, recent experimental data show that host specialization in *A. gossypii* populations is conditional. The host range expansion of host-specialized *A. gossypii* has been found under some specific conditions (Liu, Zhai & Zhang, 2008; Wu et al., 2013; Xu, Ma & Liu, 2014; Liu, Xu & Lei, 2017; Hu et al., 2017). Feeding experience on cowpea for one generation or on hibiscus plants for three generations extended the host range of the cucurbit-specialized biotype, and this biotype gained the ability to use both the cotton and cucumber seedlings (Liu, Zhai & Zhang, 2008; Wu et al., 2013). When the cotton-specialized biotype was reared for five or more generations on zucchini plants, it acquired the ability to use cucumber seedlings, but consequently lost the ability to use cotton plants (Wu et al., 2013). The cucumber at the inflorescence stage and cotton at the infructescence stage acted as a refuge for the cotton- and cucumber-specialized aphid biotypes, respectively, when these aphids encountered a food deficiency (Liu, Xu & Lei, 2017). These previous studies illustrate that the host range of aphid populations varies with the population’s feeding experience, and the host specialization of aphids to a specific host plant can change over a small number of generations.

Feeding experience, or host shifts, give aphids an opportunity to ingest some novel substances from new plants. Nutritional quality and secondary metabolites in plants affect the food quality of host plants for insect herbivores (Felton et al., 1989; Felton, 1996; Couture et al., 2016). Only the populations or species of insects adapted to the nutrients and the secondary metabolites of plants can survive and colonize the plant. That feeding experience on specific host plants shifted the host specialization of *A. gossypii* suggests that substances in host plants might determine the host range of aphids. How feeding experience over a small number of generations can maintain or change the host range of aphids is not clear.

On the other hand, genetic backgrounds of aphids may affect their host range. It has been found that the genetic structure of insect populations changed in association with the use of novel host plants (Singer, Thomas & Parmesan, 1993). High genetic differentiation between host races was found in the pea aphid (Jaquiéry et al., 2012). The main genotypes of *A. gossypii* on cotton and cucurbit plants were also different (Carletto et al., 2009; Wang et al., 2016; Liu, Xu & Lei, 2017).

In this study, we propose the hypothesis that the host range of the cotton-melon aphid is determined by aphid genetic background and feeding experience. Due to the coexistence of nutrition and defensive contents in plants, it is difficult to distinguish the roles of nutrition and secondary metabolites in aphid feeding experience. Therefore, experiments using artificial diets can reveal how feeding experience alters insect host range, because the contents in artificial diet are wholly controllable. In this study, we firstly confirmed that three aphid genotypes in *A. gossypii* populations collected from cotton did not establish populations on intact plants or excised leaves of cucumber, and the other three genotypes collected from cucumber did not establish populations on intact plants or excised leaves of cotton. Secondly we examined the performance of these six aphid genotypes on artificial diet without plant secondary metabolites, and found that all of them could complete their life cycles. And then, these six aphid genotypes reared on artificial diet were transferred onto cotton and cucumber excised leaves to examine their ability to use leaves of both plant species. Finally, the genotypes that established populations on both the cotton and cucumber leaves via feeding experience on artificial diet were transferred back onto the cotton and cucumber seedlings to examine the change in host range. The study addresses the role of feeding experience on artificial diet in determining aphid host range, and provides a better understanding of the formation of aphid host specialization.

## Materials and Methods

### Aphids and genotyping

The cotton-melon aphids, *A. gossypii*, used in this study were originally collected from cotton and cucumber fields in Nanjing, Huangshan, and Anyang, China. The collection sites for each aphid sample were at least 100 m apart to reduce the risk of sampling offspring from the same clone. The genotype of each aphid was examined by five microsatellite loci (Vanlerberghe-Masutti, Chavigny & Fuller, 1999). The six aphid genotypes used in this study were genetically distant (Table 1). Three were collected from cotton (CO-1, CO-2, CO-3), and three from cucumber (CU-1, CU-2, CU-3) (Table 1). These six aphid genotypes were reared under conditions of 27 °C, L:D = 14 h:10 h and RH = 75% using their original host plant seedlings of cotton (Sumian 4) and cucumber (Lufeng). Host plants were cultured in a greenhouse at 27 °C in the campus of Nanjing Agricultural University, Nanjing, China, from September 2016 to June 2017. Each genotype reproduced parthenogenetically throughout the rearing period. Endosymbionts in all six genotypes of aphids were examined by diagnostic PCR with specific primers based on 16S rRNA gene sequences from *Buchnera aphidicola*, *Regiella insecticola*, *Hamiltonella defense*, *Serratia symbiotica*, *Arsenophonus*, *Rickettsia*, *Rickettsiella*, *Spiroplasma*, and X type and the *wsp* gene from *Wolbachia* (Zhang et al., 2016). We found that all six aphid genotypes did not host these symbionts except *B. aphidicola*, an obligate symbiont.

**Table 1:** Collection information and genotypes of the cotton-melon aphids used in this study.

Genotype	Host plant	Sampling date	Sampling location	Microsatellite loci				
				Ago-24	Ago-53	Ago-59	Ago-66	Ago-89
CO-1	Cotton	Jul 2001	Nanjing	114 114	118 118	161 161	147 167	151 151
CO-2	Cotton	Aug 2014	Huangshan	114 153	118 160	167 182	147 167	151 163
CO-3	Cotton	Jul 2014	Nanjing	153 163	118 160	161 167	147 167	151 163
CU-1	Cucumber	Jul 2001	Nanjing	153 153	118 118	182 200	147 167	151 163
CU-2	Cucumber	Aug 2014	Nanjing	153 217	118 118	182 200	147 167	151 163
CU-3	Cucumber	Aug 2014	Anyang	114 153	118 118	161 161	147 147	151 151

### Population establishment of six aphid genotypes on the cotton and cucumber plants

Cotton and cucumber genotypes CO1-3 and CU1-3 were transferred onto cotton and cucumber plants with six leaves and reared at 27 °C and 75% RH in a laboratory growth chamber. Twenty 7-day-old apterous aphids were placed onto each individual plant, and then covered with a plastic chamber. The number of aphids (population size) in a chamber was counted every two days until all the aphids died or the aphid population increased. The death of aphids indicated that the genotype could not establish a population on the tested plant; an increase of population size indicated that the genotype could establish a population. Each aphid genotype had three replicates on both cotton and cucumber plants.

### Population establishment of six aphid genotypes on the cotton and cucumber excised leaves

Leaves cut from cotton and cucumber seedlings at the growth stage with 4–9 leaves were used to rear six aphid genotypes at 27 °C and 75% RH. The leaf petiole was wrapped in wet cotton wool to keep it fresh. One leaf was placed into a Petri dish (90 mm diameter, 20 mm height), and then 40 7-day-old apterous aphids were released into the dish. The total number of aphids in a Petri dish was investigated every three days for six time points to determine if the aphid population increased or died on the tested leaf. The leaf in a dish was replaced with a fresh one every three days. The experiment for each genotype reared on excised leaves from the natal and alternative host plants was performed with five and ten replicates, respectively.

### Performance of aphids on artificial diet and excised leaves

**Figure 1:**
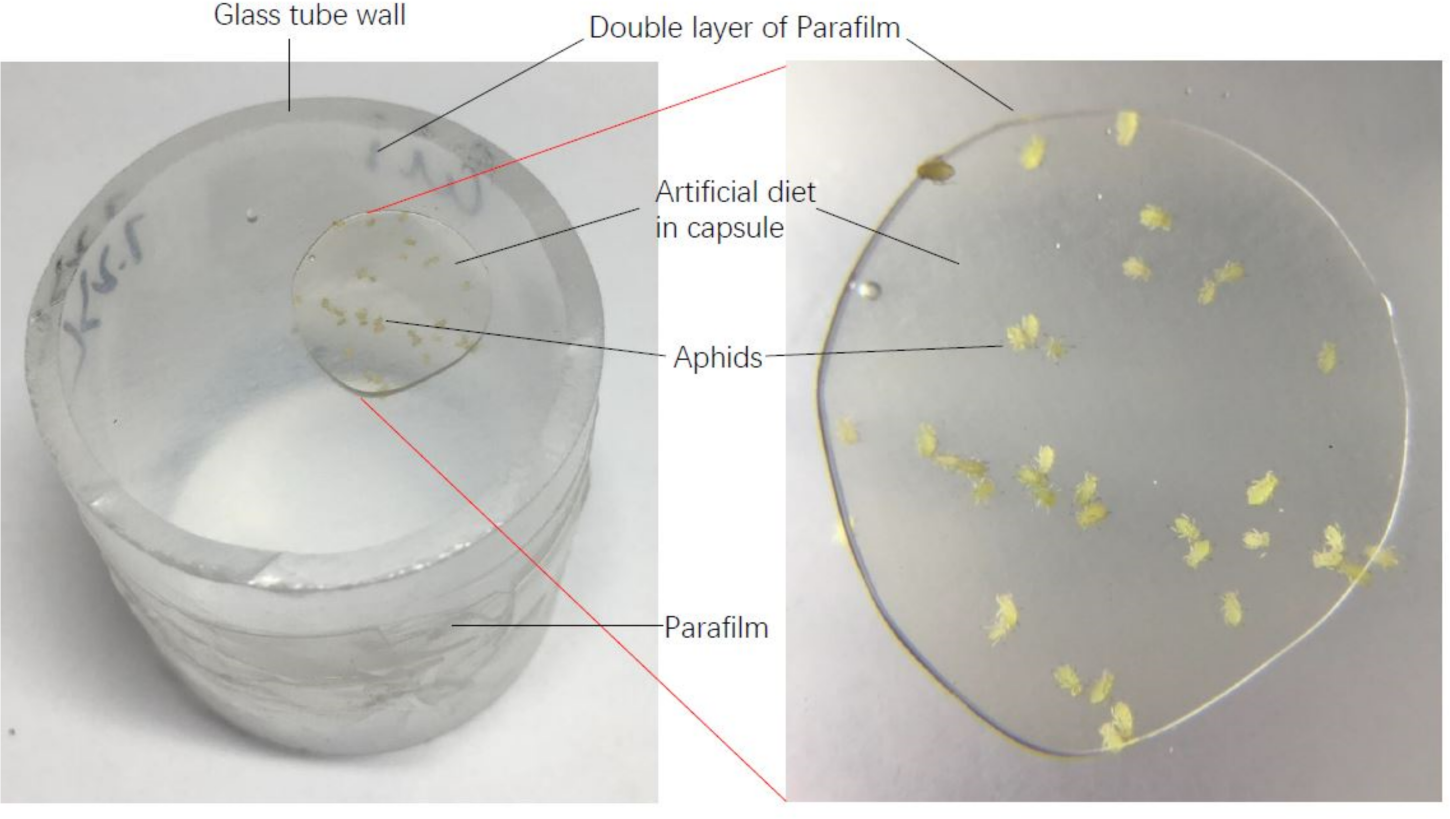
A diagram of artificial diet used in this experiment.

The liquid artificial diet of aphids was prepared according to the method by Mittler & Dadd (1962) and Auclair (1967a), Auclair (1967b). The main ingredients of this artificial diet are amino acids, sugar and vitamin B, excluding any secondary metabolites in host plants (Table 2). Life tables of each aphid genotype on the artificial diet were established. During the experiment, 150 µl artificial diet was placed between two layers of thin Parafilm which was fixed to one end of a glass tube (25 mm in diameter and 30 mm height). Fifteen to twenty apterous adult aphids were transferred onto the Parafilm in the tube, and then the other end of the tube was covered with one layer of Parafilm to prevent escape of aphids (Fig. 1). The next day, all the adults in the tube were removed, and ten newly-born nymphs were maintained as the original cohort for establishing the population documented in the life table. The survival and reproduction of the cohort of aphids were surveyed every two days until they died. During the reproductive period, nymphs were counted and then removed. The artificial diet was replaced every three days. The life tables of six aphid genotypes were performed on the artificial diet with 4 or 5 replications.

**Table 2:** Ingredient of the artificial diet in 100 ml H_2_O.

Ingredient	Dosage/mg	Ingredient	Dosage/mg
Cholesterol	5.0	Sucrose	30,000.0
L-Arginine	400.0	KH_2_PO_4_	500.0
L-Threonine	200.0	MgCl_2_⋅6H_2_O	200.0
L-Leucine	200.0	Biotin (VB_7_)	0.2
L-Tryptophane	120.0	Folic acid	0.5
L-Aspartic acid	600.0	Riboflavin (VB_2_)	2.5
Glycine	100.0	Pyridoxol (VB_6_)	2.5
L-Tyrosine	20.0	Calcium pantothenate (VB_5_)	5.0
γ-aminobutyric acid	20.0	Choline chloride	50.0
L-Histidine	200.0	Thiamine hydrochloride (VB_1_)	2.5
L-Lysine	200.0	Nicotinic acid (VB_3_)	10.0
L-Phenylalanine	120.0	Inose	50.0
L-Asparagine	110.0	P-Aminobenzoic acid	10.0
L-Glutamin	600.0	Ascorbic acid	100.0
L-Serine	100.0	Ca(H_2_PO_4_)_2_	1.4
L-Cysteine	50.0	FeC_6_H_5_O_7_⋅5H_2_O	0.4
L-Methionine	120.0	MgSO_4_	1.4
L-Valine	200.0	CuCl_2_⋅2H_2_O	0.6
L-Isoleucine	150.0	ZnCl_2_	2.6
L-Glutamic acid	200.0	Ca(C_3_H_5_O_3_)_2_⋅5H_2_O	3.4
L-Alanine	100.0	NaH_2_PO_4_⋅2H_2_O	1.0
L-Proline	100.0	NaCl	0.6
L-Cystine	10.0	MnCl_2_⋅4H_2_O	1.0

The life tables of three genotypes from cotton and three genotypes from cucumber were also performed on the excised cotton and cucumber leaves, respectively. The leaf petiole was wrapped in wet cotton wool to keep leaf fresh, and then the leaf was placed into a Petri dish. Five apterous adult aphids were transferred onto the leaf for producing offspring at night, and then the mother aphids were removed and ten new-born aphids were kept for surveying their survival and reproduction. The survey was performed every two days following the method used in the artificial diet experiment above. Five replications were performed for each genotype on the cotton and cucumber excised leaves. The intrinsic rate of natural increase (*r*_*m*_) was used to indicate the performance of aphids on artificial diet or host leaves which was calculated by the formula: *r*_*m*_ = ln(*R*_0_)/*T*, where *R*_0_= Σ *l*_*x*_ *m*_*x*_, *T* = Σ*xl*_*x*_*m*_*x*_∕*R*_0_, *l*_*x*_ is the proportion of individuals in the initial cohort alive at age *x* days, and *m*_*x*_ is the mean number of progeny produced per mother aphid alive on day *x*.

### Population growth of the artificial diet-conditioned aphids on cotton and cucumber leaves

The population growth of six aphid genotypes conditioned by artificial diet was measured on the excised cotton and cucumber leaves. Forty 7-day-old apterous aphids fed on artificial diet for seven days were transferred onto an excised cotton or cucumber leaf in a Petri dish, and then the number of aphids (population size) was observed every three days until all the aphids died or the aphid population increased. The observation lasted for at least 16 days. The experiments for all six genotypes on cotton and cucumber leaves were replicated 10 times except the genotype CU-1 on cucumber with five replications because of the lower progeny of CU-1 on artificial diet and stable survival rate on cucumber. These artificial diet-conditioned aphids could not establish populations on the alternative host plant, so the number of aphids on the intact host plant was not analyzed.

### Population establishment on cotton and cucumber plants for these aphids adapted to the alternative host leaves via artificial diet conditioning

We found that two aphid genotypes (CO-2 and CO-3) from cotton and one genotype (CU-3) from cucumber could establish populations on excised leaves of the alternative host plants, cucumber and cotton, respectively, when they fed on artificial diet for seven days. Therefore, we reared these genotypes on their alternative host plant leaves for more than 150 days (>12 generations), and then we transferred them onto an intact cotton and cucumber plant (with 4–6 leaves) to examine whether these novel leaf-conditioned aphids could use both the cotton and cucumber plants. Twenty 7-day conditioned apterous aphids were transferred from the alternative host leaves onto cotton and cucumber plants, and then the population sizes were observed every two days. The observation lasted for 17–49 days according to the rate of population increase. When the number of aphids had grown more than 10-fold on the alternative host plant, the observation ended. Three replications were performed for each genotype and host plant.

### Data analysis

The *r*_*m*_ of different aphid genotypes on artificial diet and excised leaves was analyzed using a general linear model and the aphid genotype and food type (excised leaf and artificial diet) were considered as fixed factors. The *r*_*m*_ of each genotype reared on artificial diet and excised leaves was compared using student’s *t* test. Comparisons of the *r*_*m*_ among different genotypes on artificial diet or excised leaves, and population sizes among aphid genotypes on cotton and cucumber were analyzed using ANOVA followed by Tukey HSD post hoc comparisons. All the statistical analyses were performed using software SPSS 17.

## Results

### Population establishment of different aphid genotypes on intact plants of cotton and cucumber

**Figure 2:**
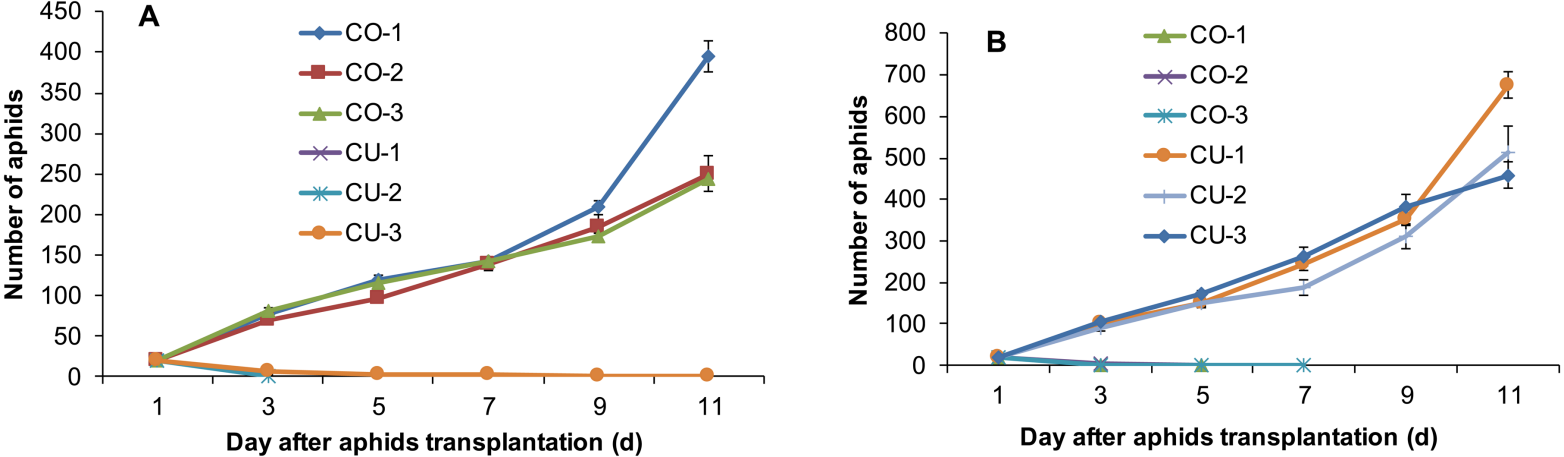
Population sizes of six aphid genotypes. (A) Aphids on cotton plants. (B) Aphids on cucumber plants.

The aphids from intact cotton and cucumber plants did not establish populations on the alternative host plant. The three aphid genotypes collected from cotton CO-1, CO-2, and CO-3 established populations on cotton plants, but the three genotypes from cucumber CU-1, CU-2, and CU-3 did not establish populations on cotton (Fig. 2A). The aphid genotypes from cucumber survived 3-11 days on cotton plants (Fig. 2A). Similarly, the three aphid genotypes collected from cucumber CU-1, CU-2, and CU-3 established populations on cucumber plants, but genotypes from cotton CO-1, CO-2, and CO-3 did not establish populations on cucumber plants, and they survived only 3–7 days (Fig. 2B).

### Population growth of different aphid genotypes on excised leaves of the alternative host plant

**Figure 3:**
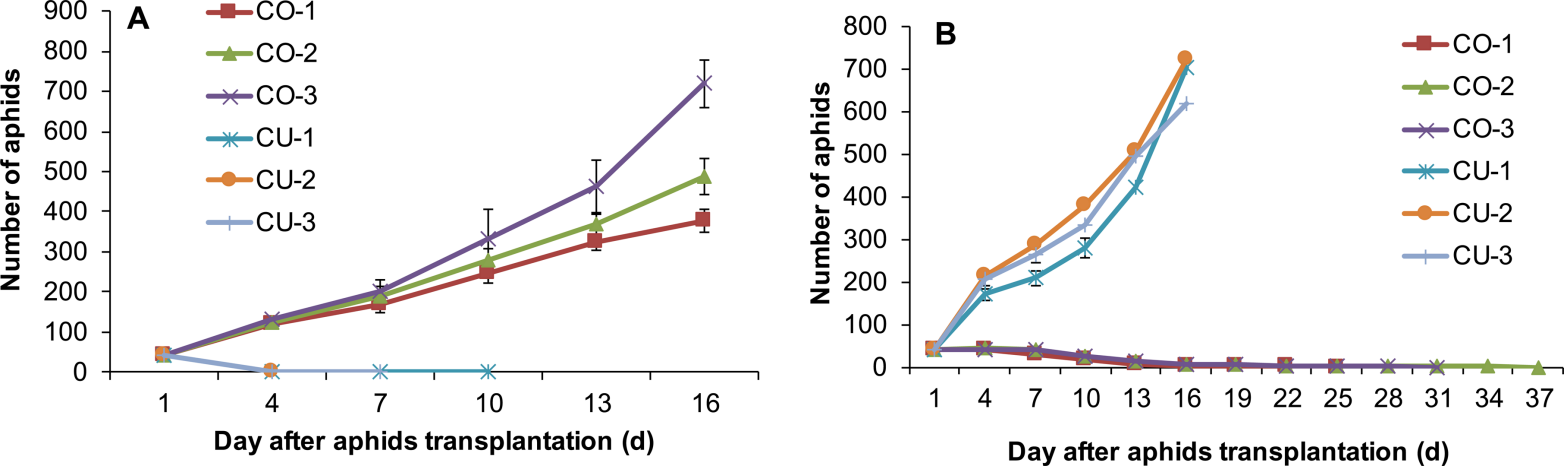
Population sizes of six aphid genotypes on excised leaves. (A) Leaves from cotton. (B) Leaves from cucumber.

The aphids on cotton and cucumber did not establish populations on the excised leaves from the alternative host plant. The aphid populations of six genotypes increased well on the excised leaves from their natal host plants (Figs. 3A 3B), but the genotypes from cucumber survived 4–10 days on the excised cotton leaves (Fig. 3A), and the genotypes form cotton survived 25–37 days on the excised cucumber leaves (Fig. 3B).

### Performance of different aphid genotypes on artificial diet

**Figure 4:**
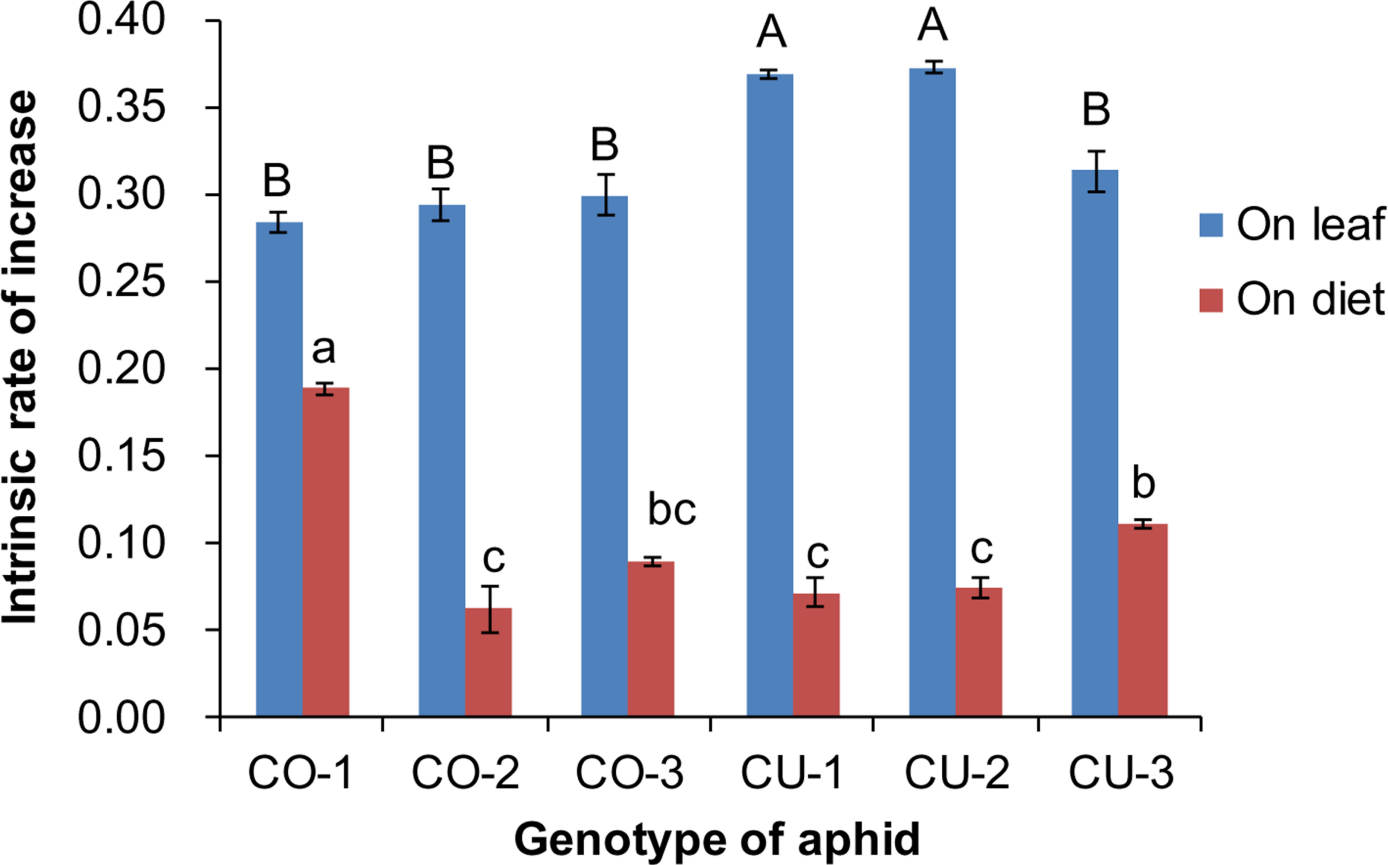
Intrinsic rate of increase (*r*_*m*_) of six aphid genotypes on artificial diet and native host leaves. Different lowercases and uppercase represent significant difference among aphid genotypes on host leaves and artificial diet, respectively at *P* < 0.05.

The artificial diet was not very suitable for the six aphid genotypes, compared to the host leaf. The intrinsic rates of natural increase (*r*_*m*_) of aphid populations were significantly affected by food type (*F*_1,59_ = 2, 316.782, *P* < 0.001) and aphid genotype (*F*_5,59_ = 14.095, *P* < 0.001) (Fig. 4). The *r*_*m*_ of all six aphid genotypes on excised leaves from the natal host plant were significantly higher than that on the artificial diet (CO-1: *t* = 14.141, *P* < 0.001; CO-2: *t* = 14.265, *P* < 0.001; CO-3: *t* = 16.483, *P* < 0.001; CU-1: *t* = 29.805, *P* < 0.001; CU-2: *t* = 42.605, *P* < 0.001; CU-3: *t* = 17.075, *P* < 0.001) (Fig. 4). The *r*_*m*_ values on natal host leaves were 1.5 to 5.2 times higher than the values on the artificial diet for all six aphid genotypes. The *r*_*m*_ of CO-1 was significantly higher on the artificial diet than that of the other five genotypes, and the *r*_*m*_ of CU-3 was significantly higher than that of CO-2, CU-1, and CU-2 on artificial diet (*F*_5,24_ = 42.634, *P* < 0.001) (Fig. 4).

### Feeding experience on artificial diet affects host range of aphids

**Figure 5:**
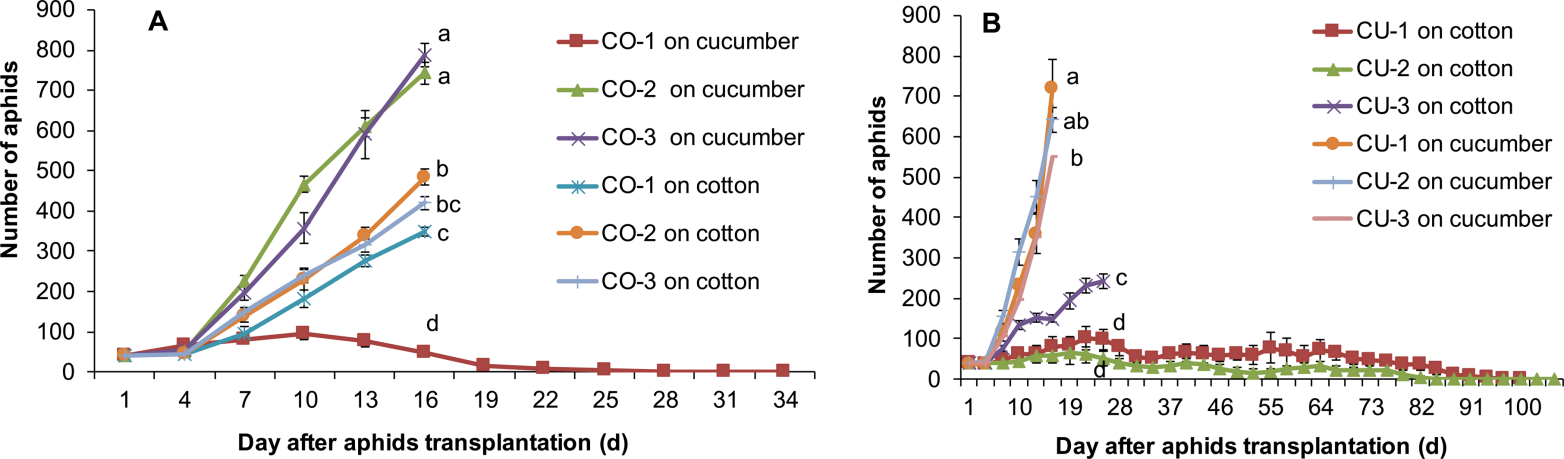
Population growth of artificial diet-conditioned aphids on cotton and cucumber excised leaves. (A) three aphid genotypes from cotton. (B) three genotypes from cucumber. Different lowercase means significant difference in population size after 16 days of aphid transplantation on leaves between genotypes on cotton and cucumber leaves at *P* < 0.05.

Feeding experience on artificial diet altered host plant range of some aphid genotypes. Three aphid genotypes from cotton CO-1, CO-2, and CO-3 still maintained the ability to use their natal host cotton leaf when they were conditioned to the artificial diet for seven days (Fig. 5A). Moreover, two aphid genotypes CO-2 and CO-3 acquired the ability to use cucumber leaf, an alternative host plant (Fig. 5A). The population sizes of CO-2 and CO-3 on excised cucumber leaves were significantly higher than that of CO-1, CO-2 and CO-3 on excised cotton leaves (*F*_5,54_ = 181.42, *P* < 0.001; Fig. 5A). Although genotype CO-1 conditioned to artificial diet could survive a long time on excised cucumber leaves (34 days), it did not establish a population (Fig. 5A).

Three aphid genotypes from cucumber CU-1, CU-2, and CU-3 established populations on excised cucumber leaves after they fed on artificial diet for seven days (Fig. 5B). Interestingly, one aphid genotype (CU-3) established a population on excised cotton leaves too after feeding experience on artificial diet, and the population size of this genotype on cotton leaves was significantly larger than those of genotypes CU-1 and CU-2 ( *F*_5,49_ = 126.91, *P* < 0.001) (Fig. 5B). Genotypes CU-1 and CU-2 could survive more than 100 days on cotton leaves after feeding experience on artificial diet, but eventually all of them died (Fig. 5B).

### Feeding experience on the excised leaf affects aphid host range

**Figure 6:**
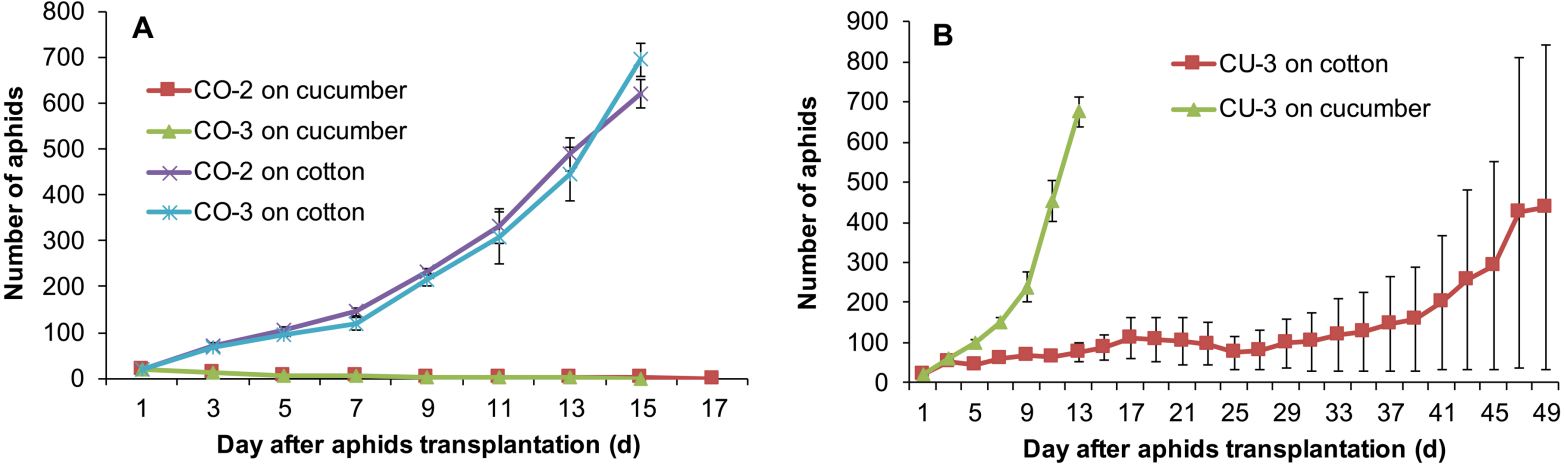
Population growth of three aphid genotypes on cotton and cucumber plants. (A) Two genotypes from cotton. (B) One genotype from cucumber. These three genotypes were reared for 12 generations on the alternative host leaves after conditioning to artificial diet for seven days.

Host range expansion of aphids mediated by feeding experience was genotype-dependent. The aphid genotypes CO-2 and CO-3 established populations on the excised leaves of cucumber after feeding experience on artificial diet. However, after they were reared for 12 successive generations on the cucumber leaves, both genotypes still could use cotton plants, but could not use cucumber plants (Fig. 6A). By contrast, the genotype CU-3 fed on excised cotton leaves for 12 generations after experience on artificial diet could use both the cucumber and cotton plants (Fig. 6B), suggesting the expansion of host range. The population growth of these CU-3 on cotton plants was faster in one replicate after 25 days than in the other two, and therefore, the average number of aphids showed a high variance (Fig. 6B).

## Discussion

Phytophagous insects, including dietary generalist species, often exhibit host specialization (Futuyma & Moreno, 1988; Jaenike, 1990; Sword, Joern & Senior, 2005; Stilmant et al., 2008). However, several factors, including both evolutionary and ecological processes, may induce changes in the host range of insect herbivores, and result in the acquisition or loss of specific host plants in their host range (West-Eberhard, 1989; Gorur, Lomonaco & Mackenzie, 2007). The host specialization of phytophagous insects is not wholly conservative (Nosil, 2002; Gorur, Lomonaco & Mackenzie, 2005; Liu, Zhai & Zhang, 2008). The results of the present study indicate that some aphid genotypes acquired the ability to survive and establish populations on excised leaves of a novel host plant which they did not use, after they underwent a feeding experience on artificial diet, but some genotypes did not acquire this ability. A previous study also found that a specific host plant could lead to a host shift of the host-specialized aphids (Wu et al., 2013). Because the experimental plants included both nutrients and secondary metabolites, it was unclear how each of these substances or if both contributed to the host shift of aphids. In this study, we found that the artificial diet lacking in secondary metabolites could alter the host range of a part of genotypes in *A. gossypii* aphids from cotton and cucumber. This result shows that feeding experience with nutrients alone can contribute to a change in host range of some aphid genotypes. Aphids feeding on different host plants may ingest different nutrients, and each genotype may adapt specifically to this nutrient profile (Via, 1991; Carletto et al., 2009; Alkhedir et al., 2016). Further study is needed to determine which specific nutrients are involved.

Plant resistance to aphids is related to host quality in terms of free amino acids composition, phytohormone content (abscisic acid, jasmonic acid, salicylic acid), and secondary metabolite profiles (Kant et al., 2004; Schilmiller & Howe, 2005; Chiozza, O’Neal & MacIntosh, 2010; Vos et al., 2013; Agut et al., 2014; Agut et al., 2016). Nutritional quality and secondary metabolite profiles vary dramatically between the natal and alternative host plants for aphids (Wang et al., 2015; Wang et al., 2017). As key nutrients for insects, free amino acids in plants are closely related to insect feeding, development, and reproduction (Mittler, 1970; Wilkinson & Douglas, 2003). Within a host plant species, phenotypic variation, sometimes characterized as plant vigor, affects the performance of aphids (Price, 1991; White, 2009); variation in plant vigor would result from variation in nutrients and secondary metabolites. In previous studies, we found that the cucurbit-specialized *A. gossypii* aphids could not survive on vigorous cotton plants, but they could colonize the old cotton plants by feeding on their old leaves. In contrast, the cotton-specialized aphids preferentially chose the vigorous seedlings of cotton and fed on the new leaves (Xu, Ma & Liu, 2014; Liu, Xu & Lei, 2017). In this study, the results indicate that the excised leaves and intact plants have different effects on performance of aphids. Other studies point to some of the specific differences between excised leaves and intact plants. The excised leaves of corn seedlings produced more jasmonic acid- and volicitin-induced sesquiterpene volatile than intact plants (Schmelz, Alborn & Tumlinson, 2001). Leaf excision stimulated increased ethylene production in rice leaves (Tsai, Hung & Kao, 1996). The amino acid content in the excised turnip leaves was higher than that in the intact leaves (Thomas, Sorensen & Painter, 1966). Plant secondary metabolites and nutrients have a strong impact on consumption and growth in dietary generalist herbivores, such as the cabbage looper *Trichoplusia ni* (Kaplan, McArt & Thaler, 2014). Excised leaves and old plants might have reduced defensive traits and improved nutrients to aphids compared to vigorously growing tissues, so aphids from other hosts might survive on them. For example, survival of spotted aphids was higher on excised than intact leaves of alfalfa (Thompson, Stewart & Morris, 1966). However, in this study, we also found that an artificial diet lacking secondary metabolites not only changed performance of aphids, but also induced host range expansion of these aphids. The result indicates that just host nutrients and not the secondary metabolites which aphids ingest from food can affect aphid host range. We speculated that the cucurbit-specialized and cotton-specialized biotypes in *A. gossypii* populations might result from different nutrient profiles, such as amino acids, in cotton and cucumber plants. Better understanding of the relationships between plant nutrients and the host range of herbivorous insects will inform the physiological mechanism by which feeding experience alters host range shifts in *A. gossypii*.

In this study, we also found that the effect of feeding experience on aphid host range was dependent on aphid genotypes. Feeding experience induced the expansion of host range in some aphid genotypes but not all. Aphids with different genetic backgrounds may have different requirements for nutrition, and consequently live on different host plants. Therefore, the nutrients which can induce changes in host range for different genotypes may be different. It has been found that feeding experience of two clones of the pea aphid, *Acyrthosiphon pisum*, on alfalfa or red clover did not alter aphid’s specialization to the natal host plant (Via, 1991), and some genotypes of *Rhopalosiphum maidis* could withstand wheat stressing conditions, but the conditioning on wheat did not decrease the capacity to use their native host Johnson grass (Caballero, Ramirez & Niemeyer, 2001). Genetic differentiations may contribute to host shifts of insects. The rapid adaptation of seed beetle *Callosobruchus maculatus* to a poor host was mediated by genetic variation at multiple genetic loci, and the contribution of genetic drift was small (Gompert & Messina, 2016). Genetic trade-offs associated with host use have been found in phytophagous insects (Hawthorne & Via, 2001; Gompert & Messina, 2016). Genetic structures of *A. gossypii* populations on cotton and cucumber plants were different (Liu, Xu & Lei, 2017). Feeding on a constant set of host plants in the life cycle of aphids may result in the genetic differentiation among different host strains, causing the formation of host specialization and evolution of host plant use. Therefore, using genomics techniques will clarify the molecular mechanisms of evolutionary adaptation of insect herbivores to their host plants.

Epigenetic effects and microbial symbionts cannot be ignored in changes of aphid host range (Kim, Thairu & Hansen, 2016). DNA methylation is a general epigenetic mechanism for organisms to adapt to environmental changes. For example, two biotypes of the Russian wheat aphid, *Diuraphis noxia*, using different host plants showed different methylation levels (Gong et al., 2012). The high variance in the population size of CU3 conditioned by artificial diet and the alternative host plant appeared on cotton plants, and only some aphid genotypes, but not all, could change their host range via the feeding experience on artificial diet. These results imply that there may be epigenetic effects of aphid host range mediated by feeding experience. On the other hand, insect symbionts may be hidden players in insect–plant interactions (Frago, Dicke & Godfray, 2012). Host plant specialization of the pea aphids was found to be governed by a facultative symbiont, *R. insecticola* (Tsuchida, Koga & Fukatsu, 2004). Although all the aphid genotypes used in this study were not infected with facultative symbionts, the obligate symbiont *B. aphidicola* was present. The *B. aphidicola* can supply nutrients for aphids, such as amino acids (Douglas & Prosser, 1992), and changes in the population size of this symbiont can affect fitness of aphids (Chen, Lai & Kuo, 2009; Vogel & Moran, 2011). Symbiont density in aphids was different when aphids fed on different host plants (Zhang et al., 2016). Insect symbionts contributed to a variety of extended insect host phenotypes (Kim, Thairu & Hansen, 2016). We speculate that different dietary nutrients may affect the changes of aphids in epigenomics or microbial symbionts, and consequently results in the change of aphid host range.

## Conclusions

The cotton-melon aphid populations exhibit obvious host specialization to cotton and cucumber plants. The aphids on cotton do not use cucumber, and aphids on cucumber cannot establish populations on cotton. Aphids from both the cotton and cucumber can establish populations on an artificial diet, and the short-term feeding experience on this artificial diet results in host expansion of some genotypes. The host range change of aphids mediated by feeding experience on artificial diet is dependent on aphid genetic, epigenetic, or symbiont backgrounds. Nutrients of host plants may contribute to the formation of host specialization and evolution of host plant use in aphid populations. Host specialization may be easy to form in aphid populations feeding on a constant set of host plants in life cycle.

##  Supplemental Information

10.7717/peerj.7774/supp-1 Supplemental Information 1 Raw data for Figs. 1–5

## Additional Information and Declarations

### Data Availability

The following information was supplied regarding data availability: The raw data are available as a Supplemental File.
